# Experimental sleep disruption attenuates morphine analgesia: findings from a randomized trial and implications for the opioid abuse epidemic

**DOI:** 10.1038/s41598-020-76934-1

**Published:** 2020-11-18

**Authors:** Michael T. Smith, Chung Jung Mun, Bethany Remeniuk, Patrick H. Finan, Claudia M. Campbell, Luis F. Buenaver, Mercedes Robinson, Brook Fulton, David Andrew Tompkins, Jean-Michel Tremblay, Eric C. Strain, Michael R. Irwin

**Affiliations:** 1 Division of Behavioral Medicine, Department of Psychiatry and Behavioral Sciences, Johns Hopkins University, School of Medicine, 5510 Nathan Shock Drive, Suite 100, Baltimore, MD 21225 USA; 2 Virginia Tech Carilion School of Medicine, Roanoke, VA 24016 USA; 3 Department of Psychiatry, UCSF School of Medicine, San Francisco, CA 94110 USA; 4 Edgybees Inc., Gaithersburg, MD 20878 USA; 5 Cousins Center for Psychoneuroimmunology, UCLA Semel Institute for Neuroscience and Human Behavior, Los Angeles, CA 90024 USA; 6 Department of Psychiatry and Biobehavioral Sciences, David Geffen School of Medicine At UCLA, Los Angeles, CA 90095 USA

**Keywords:** Psychology, Medical research, Risk factors

## Abstract

Preclinical studies demonstrate that sleep disruption diminishes morphine analgesia and modulates reward processing. We sought to translate these preclinical findings to humans by examining whether sleep disruption alters morphine’s analgesic and hedonic properties. We randomized 100 healthy adults to receive morphine versus placebo after two nights of undisturbed sleep (US) and two nights of forced awakening (FA) sleep disruption. Sleep conditions were counterbalanced, separated by a two-week washout. The morning after both sleep conditions, we tested cold pressor pain tolerance before and 40-min after double-blind injection of .08 mg/kg morphine or placebo. The primary outcome was the analgesia index, calculated as the change in cold pressor hand withdrawal latency (HWL) before and after drug injection. Secondary outcomes were ratings of feeling “high,” drug “liking,” and negative drug effects. We found a significant sleep condition by drug interaction on the analgesia index (95% CI − 0.57, − 0.001). After US, subjects receiving morphine demonstrated significantly longer HWL compared to placebo (95% CI 0.23, 0.65), but not after FA (95% CI − 0.05, 0.38). Morphine analgesia was diminished threefold under FA, relative to US. After FA, females (95% CI − 0.88, − 0.05), but not males (95% CI − 0.23, 0.72), reported decreased subjective “high” effects compared to US. After FA, females (95% CI 0.05, 0.27), but not males (95% CI − 0.10, 0.11), administered morphine reported increased negative drug effects compared to US. These data demonstrate that sleep disruption attenuates morphine analgesia in humans and suggest that sleep disturbed males may be at greatest risk for problematic opioid use.

## Introduction

In the US, opioid overdoses recently surpassed road accidents as the leading cause of accidental death^1^. Although the causes of the global opioid crisis are complex, over-prescription for chronic pain is a widely recognized contributor^2,3^. Nearly a third of chronic pain patients misuse (e.g., taking higher doses than prescribed) or abuse (e.g., non-medical use) opioids^4^. Mu-opioid receptor (MOR) agonist analgesics, of which morphine is prototypical, have several properties that contribute to their increased abuse potential in patients with chronic pain. Not only do they relieve severe pain, but they also have subjective euphoric effects and induce physiological dependence, especially at higher doses. Given the substantial societal and personal costs of the opioid crisis, identification of factors that influence analgesic efficacy and subjective drug effects that contribute to abuse liability is critical for prevention efforts. Targeting modifiable risk factors that both attenuate analgesia and alter the hedonic properties of opioids may be especially important in the context of chronic pain as users may escalate dosing in order to achieve adequate pain relief and improve mood^5^.

Sleep disturbance, which is reported by the vast majority (50% to 88%) of chronic pain patients, is a well-established risk factor for chronic pain^6^, and a highly prevalent and modifiable candidate risk factor for opioid misuse and abuse^7^. Pre-clinical studies demonstrate that sleep deprivation both attenuates the antinociceptive effects of morphine^8–10^, and increases sucrose and cocaine self-administration, seeking behavior, and reward value in rodents^11–13^. Although human data are limited, a self-reported night of poor sleep predicts next day increases in opioid dose among burn injury survivors^14^. Experimental evidence indicates that nociception not only disrupts sleep, but sleep loss also enhances pain sensitivity^6,15,16^. Heightened pain may in turn trigger opioid self-medication and dose escalation^17^. In addition to exacerbating pain, sleep deprivation also amplifies brain reactivity to rewards^18^ and alters downstream (MOR-dependent) dopamine signaling in the mesolimbic reward system^19^.

Whether sleep disruption influences the analgesic efficacy of MOR agonists or alters the subjective hedonic and negative drug effect indicators of abuse liability in humans remains unknown. This gap in knowledge is striking given evidence that high dosing is one of the most robust predictors of negative opioid-related outcomes, including developing opioid dependence and fatal overdoses^20,21^. Consequently, the primary objective of this study was to determine experimentally whether a sleep disruption pattern (i.e., multiple, prolonged awakenings with curtail sleep duration^22^), most commonly experienced by patients with chronic pain, reduces the analgesic effects of morphine. We secondarily sought to evaluate whether experimental sleep disruption alters subjective drug effects, including ratings of “drug liking” and “feeling high,” standard measures used in determining abuse liability^23,24^, as well as negative opioid agonist effects. Finally, we explored the possibility that sex may moderate relationships between sleep disruption, opioid analgesia and drug effect ratings, given data suggesting that females are more responsive to opioid analgesia, but have more negative side effects^25^.

## Results

**Figure 1:**
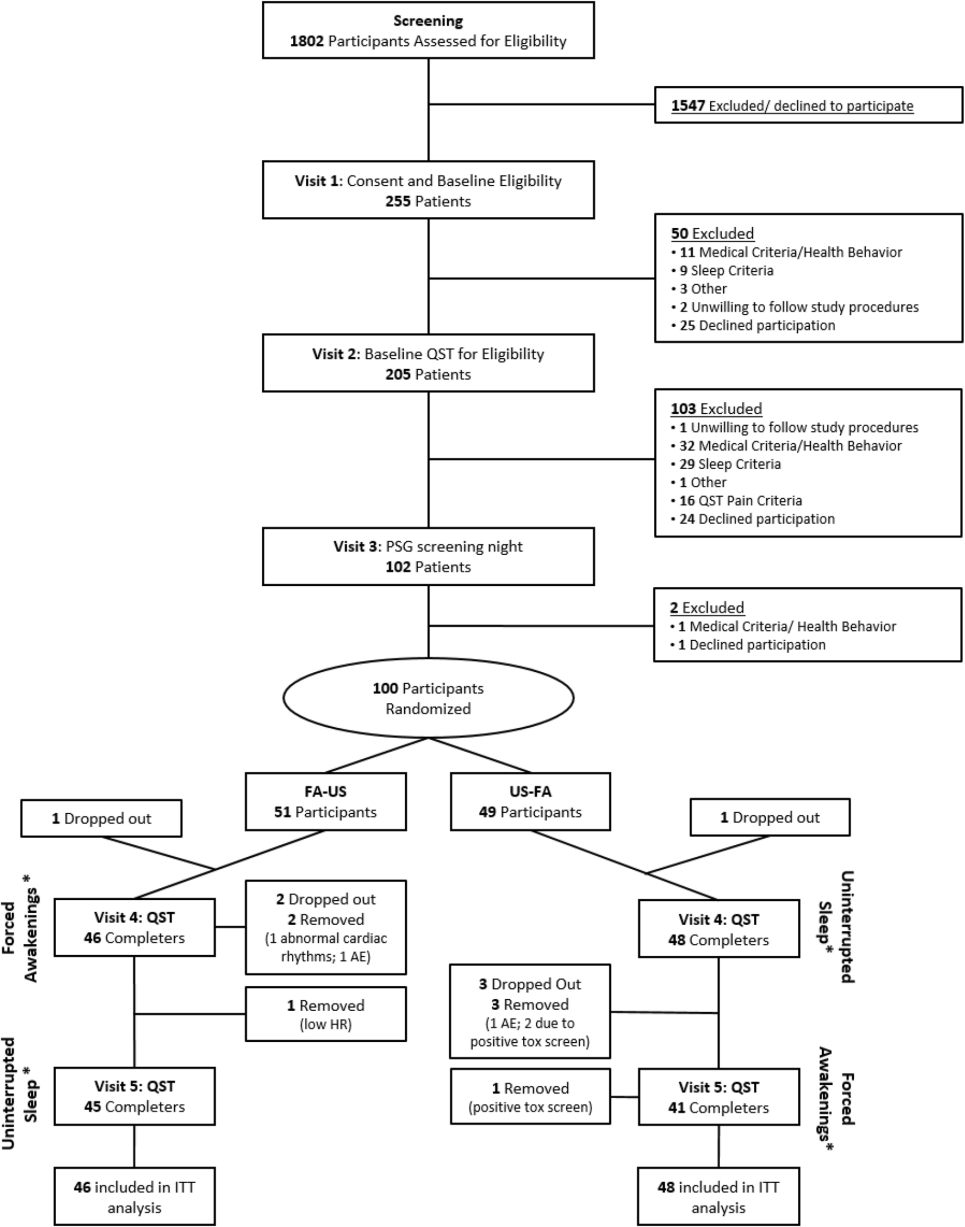
Study consort diagram. Note. QST = Quantitative Sensory Testing, PSG = Polysomnography, AE = Adverse Event, HR = Heart Rate, ITT = Intention-to-Treat; * = participants underwent two consecutive nights of the respective sleep condition prior to QST testing at each visit.

Study enrollment began June 3rd, 2013 and was completed on January 18th, 2018. A consort diagram of participant enrollment and attrition at each study visit is depicted in Fig. 1. A total of 1,802 participants were assessed for eligibility and 100 healthy, good-sleeping adults were initially randomized. A total of six participants were excluded from analyses because they either dropped out or were removed from the study prior to or during Visit 4 (first experimental visit). We investigated whether there was any systematic bias in those who were not included in the analyses. Chi-square and t-tests revealed no statistically significant (*p*-values ranging from 0.16 to 0.93) differences in baseline participant characteristics (i.e., age, sex, race, education, and employment status) between those who either dropped out prior to providing any data or were removed versus those included in the final sample.

### Sample demographics

Table 1 summarizes sample demographics by drug randomization assignment, demonstrating balance between groups on demographic characteristics. The overall mean age was 28.06 years, and close to 60% were females. Participants were racially diverse (45% Caucasian, 37% African American, and 18% other, including Asian and mixed race). Among participants, 17% described themselves as of Hispanic origin. The majority of participants had at least some college education and were currently either working or a full-time student.

**Table 1:** Demographics at baseline.

	**Total** (n = 100)	**Morphine** (n = 54)	**Placebo** (n = 46)	***p-value***
Age (Mean ± SD)	28.06 ± 6.82	28.50 ± 6.93	27.54 ± 6.73	0.48
**Sex, n (%)**				0.35
Female	58 (58.0%)	29 (53.7%)	29 (63.0%)	
**Race, n (%)**				0.42
Caucasian	45 (45.0%)	24 (44.4%)	21 (45.7%)	
African American	37 (37.0%)	20 (37.0%)	17 (37.0%)	
Asian	11 (11.0%)	4 (7.4%)	7 (15.2%)	
More than one race	3 (3.0%)	3 (5.6%)	0 (0%)	
Other/decline to state	4 (4.0%)	3 (5.6%)	1 (2.2%)	
**Ethnicity, n (%)**				0.13
Hispanic	17 (17.0%)	12 (22.2%)	5 (10.9%)	
**Education, n (%)**				0.27
High school/GED	6 (6.0%)	4 (7.5%)	2 (8.3%)	
Some college/current student	27 (27.0%)	9 (17.0%)	18 (39.2%)	
College graduate	45 (45.0%)	27 (50.9%)	18 (39.1%)	
Technical school graduate	1 (1.0%)	1 (1.9%)	0 (0.0%)	
Advanced degree	20 (20.0%)	12 (22.7%)	8 (17.4%)	
**Employment status, n (%)**				0.07
Student	35 (35.0%)	22 (41.5%)	13 (28.2%)	
Employed	48 (48.0%)	21 (39.6%)	27 (58.7%)	
Unemployed	15 (15.0%)	10 (18.9%)	5 (10.9%)	
Homemaker	1 (1.0%)	0 (0%)	1 (2.2%)	

Values represent mean (percent of total) for demographics of participants. T-test (for continuous variables) and chi-square (categorical variables) test was used to examine differences in morphine vs. placebo conditions.

### Manipulation check: effects of sleep disruption on polysomnography (PSG) sleep parameters and psychomotor vigilance

The forced awakening (FA) sleep disruption condition demonstrated significantly shorter minutes of total sleep time averaged across both nights [M = 263.20 (26.99)] compared to the undisturbed sleep (US) condition [M = 438.94 (42.60); *p* < 0.001]. Online Supplement Table 1 details PSG sleep architecture and continuity parameters across both nights for each condition. The degree of sleep loss by sleep stage and degree of disruption in sleep continuity parameters is comparable with prior studies^26^. Sleep disruption impaired Psychomotor Vigilance Task performance, with the reciprocal reaction time significantly reduced [M_US_ = 3.36 (0.06) vs. M_FA_ = 3.23 (0.06); *p* = 0.006] and the number of lapses significantly greater [M_US_ = 6.53 (1.05) vs. M_FA_ = 8.84 (1.06); *p* = 0.017], following FA compared to US.

### Primary outcome: sleep disruption effects on morphine analgesia

**Figure 2:**
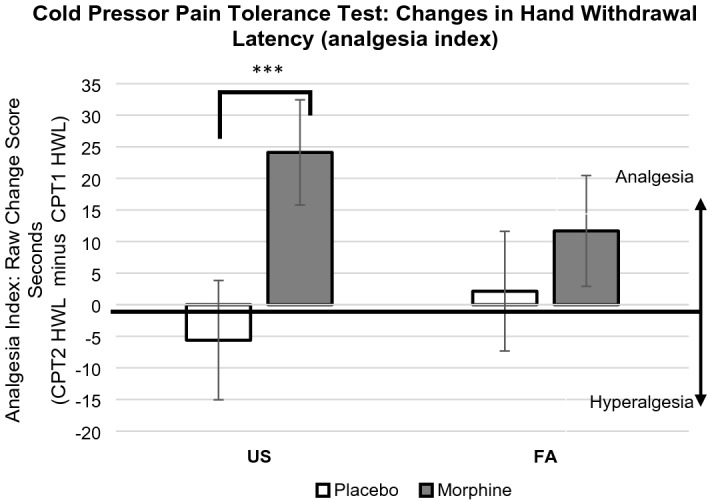
Sleep disruption effects on morphine analgesia. Note. Graph depicts raw scores but pairwise comparisons were conducted using log-transformed scores. Positive values indicate analgesia and negative values indicate hyperalgesia. *** *p* < 0.001. Key: Analgesia index = Cold pressor test hand withdrawal latency 40 min after injection (CPT2) minus cold pressor test hand withdrawal latency immediately before injection (CPT1). US = Undisturbed Sleep condition; FA = Forced Awakenings sleep disruption condition; HWL = Hand Withdrawal Latency (minutes); CPT1 = Cold pressor test immediately prior to injection; CPT2 = Cold pressor test 40 min after injection.

The two-way interaction effect of Sleep × Drug conditions on the analgesia index was statistically significant (*p* = 0.049, 95% CI − 0.57, − 0.001), controlling for baseline pressor hand withdrawal latency (HWL) scores and covariates. Detailed mixed effects regression estimates are summarized in Table 2 and the means and standard errors for the analgesia index by sleep and drug conditions are displayed in Fig. 2. See the Online Supplement Table 2 for mean(SD) HWLs at pre- and post-injection, separated by conditions. Pairwise comparisons revealed that following the US condition, participants administered morphine exhibited greater analgesia (i.e., a significant change in HWL) than those administered placebo (raw mean difference = 28.86 s; mean log transformed difference = 0.46, *SE* = 0.11, Sidak-adjusted *p* < 0.001, 95% CI 0.26, 0.66). Under the FA sleep condition, however, the analgesia index scores between morphine vs. placebo conditions were not statistically significant (raw mean difference = 9.56 s; mean log transformed difference = 0.17, *SE* = 0.10, Sidak-adjusted *p* = 0.10, 95% CI − 0.04, 0.38). Further inspection of the data showed that the magnitude of analgesic efficacy in FA condition was about threefold lower than that of US condition. Significant main effects were also found including the drug condition (*p* < 0.001, 95% CI 0.26, 0.66), age (*p* = 0.023, 95% CI 0.002, 0.02), and baseline diastolic blood pressure (*p* = 0.007, 95% CI − 0.04, − 0.01). These results indicate that participants in the morphine condition who are older, or had lower baseline diastolic blood pressure, demonstrated greater analgesia.

**Table 2:** Fixed-effect estimates in both primary and secondary outcomes.

Parameters	Changes in hand withdrawal latency					Feeling “high”				
	*B*	*SE*	*t*	*p*	95% CI	*B*	*SE*	*t*	*p*	95% CI
Intercept	0.39	0.51	0.76	0.451	[− 0.63, 1.41]	3.02	1.55	1.95	0.054	[− 0.05, 6.09]
Sleep condition × drug condition	− 0.29	0.15	− 1.98	0.049	[− 0.57, − 0.001]	0.38	0.33	1.16	0.250	[− 0.27, 1.02]
Sleep condition	0.13	0.11	1.25	0.214	[− 0.08, 0.34]	− 0.38	0.24	− 1.57	0.121	[− 0.85, 0.10]
Drug condition	0.46	0.10	4.57	< 0.001	[0.26, 0.66]	1.67	0.30	5.62	< 0.0001	[1.08, 2.26]
Race (white vs. black)	− 0.16	0.09	− 1.75	0.081	[− 0.34, 0.02]	0.23	0.30	0.76	0.450	[− 0.37, 0.82]
Race (white vs. others)	− 0.004	0.10	− 0.04	0.967	[− 0.20, 0.19]	0.11	0.34	0.31	0.757	[− 0.58, 0.79]
Sex	0.09	0.09	0.97	0.331	[− 0.09, 0.28]	0.29	0.30	0.97	0.333	[− 0.30, 0.89]
Menstrual cycle	− 0.02	0.10	− 0.17	0.869	[− 0.21, 0.18]	0.08	0.25	0.31	0.760	[− 0.41, 0.56]
Age	0.01	0.01	2.29	0.023	[0.002, 0.02]	0.00	0.02	− 0.14	0.890	[− 0.04, 0.04]
BMI	0.02	0.01	1.85	0.065	[− 0.001, 0.04]	− 0.05	0.03	− 1.61	0.110	[− 0.12, 0.01]
Baseline systolic blood pressure	0.01	0.005	1.06	0.292	[− 0.004, 0.02]	− 0.03	0.01	− 2.29	0.023	[− 0.06, − 0.004]
Baseline diastolic blood pressure	− 0.02	0.01	− 2.73	0.007	[− 0.04, − 0.01]	0.04	0.02	1.58	0.116	[− 0.01, 0.08]
Baseline hand withdrawal latency	− 0.12	0.03	− 3.89	< 0.001	[− 0.19, − 0.06]	N/A				

	Drug “liking”					Negative opioid effects				
Intercept	0.85	1.45	0.58	0.559	[− 2.02, 3.71]	2.00	0.25	8.05	< 0.001	[1.51, 2.49]
Sleep condition × drug condition	0.23	0.31	0.74	0.463	[− 0.39, 0.85]	0.07	0.06	1.21	0.231	[− 0.05, 0.19]
Sleep condition	− 0.12	0.23	− 0.51	0.611	[− 0.57, 0.34]	0.001	00.04	0.03	0.979	[− 0.09, 0.09]
Drug condition	1.26	0.28	4.52	< 0.001	[0.71, 1.81]	0.15	0.05	3.21	0.002	[0.06, 0.25]
Race (white vs. black)	− 0.37	0.28	− 1.35	0.179	[− 0.92, 0.17]	0.03	0.05	0.65	0.515	[− 0.06, 0.12]
Race (white vs. others)	0.02	0.32	0.07	0.947	[− 0.61, 0.65]	0.02	0.05	0.46	0.644	[− 0.08, 0.13]
Sex	0.08	0.28	0.29	0.769	[− 0.47, 0.63]	0.08	0.05	1.69	0.093	[− 0.01, 0.17]
Menstrual cycle	− 0.01	0.24	− 0.06	0.952	[− 0.48, 0.45]	0.01	0.04	0.26	0.796	[− 0.07, 0.10]
Age	0.01	0.02	0.38	0.702	[− 0.03, 0.04]	− 0.001	0.003	− 0.47	0.636	[− 0.01, 0.01]
BMI	− 0.06	0.03	− 1.95	0.054	[− 0.12, 0.001]	− 0.02	0.00	− 3.31	0.001	[− 0.03, − 0.01]
Baseline systolic blood pressure	− 0.01	0.01	− .67	0.502	[− 0.03, 0.02]	0.000	0.002	0.09	0.926	[− 0.004, 0.005]
Baseline diastolic blood pressure	0.03	0.02	1.45	0.150	[− 0.01, 0.07]	0.002	0.004	0.52	0.605	[− 0.006, 0.009]

### Secondary outcome: sleep disruption effects on hedonic responses to morphine

As shown in Table 2, there was no significant two-way interaction of Sleep × Drug Condition on either feeling “high” (*p* = 0.25, 95% CI − 0.27, 1.02), or drug “liking” (*p* = 0.46, 95% CI − 0.39, 0.85). However, participants who received morphine reported greater feelings of “high” (*p* < 0.001, 95% CI 1.08, 2.26) and drug “liking” (*p* < 0.001, 95% CI 0.71, 1.81) when compared to those receiving placebo.

### Secondary outcome: sleep disruption effects on subjective negative opioid effects

As indicated in Table 2, there was no significant two-way interaction of Sleep × Drug Conditions on negative effects of morphine (*p* = 0.23, 95% CI − 0.05, 0.19). However, participants who received morphine reported greater negative opioid agonist effects (*p* = 0.002, 95% CI 0.06, 0.25), compared to those receiving placebo.

### Exploratory analysis: moderating effects of sex on primary and secondary outcomes

With respect to the primary outcome, analgesia index, no significant sex moderation effects were found: Sleep Condition × Drug Condition × Sex (*B* = 0.02, *SE* = 0.29, *p* = 0.95, 95% CI − 0.56, 0.60); Sleep Condition × Sex (*B* = 0.19, *SE* = 0.15, *p* = 0.20, 95% CI − 0.11, 0.49).

**Figure 3:**
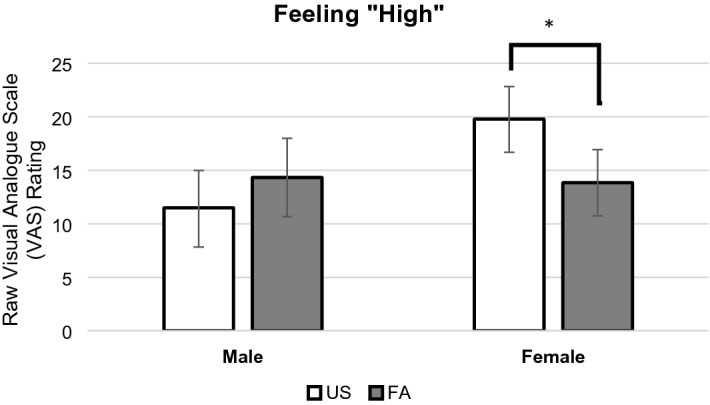
Sleep disruption effects on feeling “high” moderated by sex. Note*.* Analysis revealed a significant Sleep Condition × Sex interaction. There was no significant sex main effect. Our graph is based upon raw scores but pairwise comparisons were conducted using log-transformed scores. The VAS scale ranges from 0 to 100 with higher scores indicating more intense feeling of “high.” * *p* < 0.05. Key: US = Undisturbed Sleep condition; FA = Forced Awakenings sleep disruption condition.

In terms of secondary outcome of hedonic responses, we did not find significant three-way interactions on both feeling “high” (*B* = 0.04, *SE* = 0.64, *p* = 0.96, 95% CI − 1.25, 1.31) and drug “liking” outcomes (*B* = -0.29, *SE* = 0.63, *p* = 0.65, 95% CI − 1.54, 0.96). However, a significant two-way interaction effect of Sleep Condition × Sex on feeling “high” (*B* = -0.70, *SE* = 0.31; *p* = 0.028, 95% CI − 1.33, − 0.08) was found. As shown in Fig. 3, the pairwise comparison revealed that regardless of drug condition, under FA, females reported significantly lower levels of feeling “high” when compared to the US condition (raw mean difference = -5.94, log mean difference = -0.47, *SE* = 0.21, *p* = 0.025, 95% CI − 0.88, − 0.06). Males, however, did not show a significant difference in the level of feeling “high” between FA and US conditions (raw mean difference = 2.83, log mean difference = 0.23, *SE* = 0.24, *p* = 0.33, 95% CI − 0.24, 0.71). We did not find a significant two-way (Sleep Condition × Sex) interaction on drug “liking” outcome (*B* = -0.30, *SE* = 0.31, *p* = 0.34, 95% CI − 0.91, 0.32).

**Figure 4:**
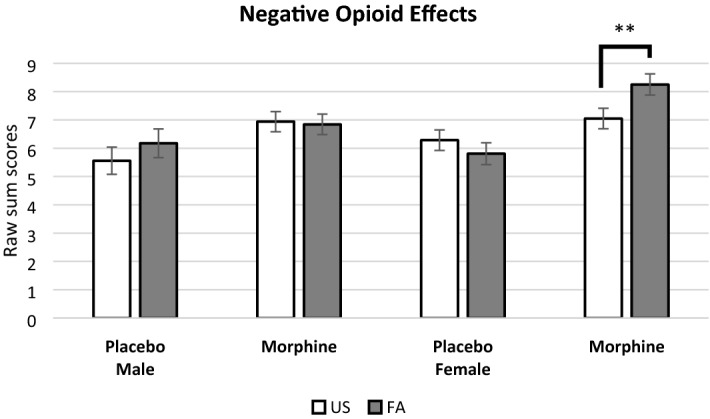
Sleep disruption and drug effect on negative opioid effects moderated by sex. Note*.* There was a three-way interaction between Sleep Condition × Drug Condition × Sex. Our graph is based upon raw scores but pairwise comparisons were conducted using log-transformed scores. The negative opioid effect sum scores range from 1 to 25 with higher values indicating greater negative opioid effects. ** *p* < 0.01. Key: US = Undisturbed Sleep condition; FA = Forced Awakenings sleep disruption condition.

With respect to secondary outcomes of negative opioid agonist effects, a significant three-way interaction of Sleep Condition × Drug × Sex was found (*B* = 0.35, *SE* = 0.12, *p* = 0.005, 95% CI 0.11, 0.56) (see Fig. 4). Pairwise comparisons revealed that females who received morphine reported increased negative opioid effects following FA, compared to US (raw mean difference = 1.20, log mean difference = 0.16, *SE* = 0.05, *p* = 0.004, 95% CI 0.05, 0.26). On the other hand, males did not report greater negative effects of morphine following FA, compared to the US condition (raw mean difference = -0.10, log mean difference = -0.01, *SE* = 0.05, *p* = 0.82, 95% CI − 0.12, 0.09).

### Adverse events

A summary of adverse events (AE) can be found in Online Supplement Table 3. The most commonly reported AEs associated with morphine administration were nausea and emesis. Females trended towards reporting more AEs overall compared to males [χ^2^(1) = 3.79, *p* = 0.052]. When AEs were limited to participants assigned to morphine, females exhibited a significantly greater number of AEs compared to males [χ^2^(1) = 11.49, *p* = 0.001]. The number of adverse events were not associated with sleep condition [χ^2^(1) = 1.11, *p* = 0.29].

**Table 3:** Inclusion and exclusion criteria.

**Inclusion criteria**
Healthy, 18–48 years old meeting Research Diagnostic Criteria for Normal Sleepers
Non-smoker/nicotine user
Low caffeine users (≤ 2 cups of coffee or equivalent per day)
Stable sleep phase within 21:00 and 10:00
Pittsburgh Sleep Quality Index Total Score < 5
Total sleep time between 6.5 and 8.5 h/night; sleep efficiency ≥ 85%; Epworth Sleepiness Scale < 10 (confirmed with averages of 1 week of sleep diary monitoring)
**Exclusion criteria**
BMI ≥ 35
History of chronic pain (lifetime history of pain persisting for ≥ 6 months)
Acute pain (measured via McGill Pain Questionnaire and 2 weeks of sleep diaries)
Significant medical/psychiatric morbidity within 6 months or lifetime history of: bipolar disorder, psychotic disorder, recurrent major depression, posttraumatic stress disorder, or seizures
Respiratory, hepatic, renal or cardiac conditions contraindicating opioid administration
Lifetime history of substance abuse or dependence, including alcohol; opioid use > 36 doses or > 7 days consecutive use
Prior adverse reactions to general anesthetics/opioids or capsaicin
Clinically significant abnormal complete blood count or comprehensive metabolic profile
Positive toxicology screen for recreational drugs of abuse, stimulants, opioids or benzodiazepines
Pregnant or lactating females
Polysomnography—confirmed apnea–hypopnea index < 10/h
Polysomnography—confirmed Period Periodic limb movements with arousal > 15/h
Lifetime history of serious head injury that is judged to influence pain processing or sleep
Pretesting capsaicin pain rating < 15/100 or > 85/100

## Discussion

The principal finding of this study is that two nights of experimental sleep disruption, similar to the objective sleep patterns most commonly observed in chronic pain (prolonged multiple awakenings with curtailed sleep time)^22^, attenuated next day morphine analgesia in young healthy adults. After US, morphine yielded robust and significant analgesic effects compared to placebo. After disrupted sleep, however, morphine yielded threefold less and statistically non-significant analgesic effects, compared to placebo. Additionally, we found an effect of sleep disruption on the propensity to feel “high” following drug administration that was moderated by sex, such that females, but not males, reported feeling less “high” from both morphine and placebo after sleep disruption, compared to US. Females, administered morphine, also reported greater negative opioid agonist effects such as feeling “heavy or sluggish” or having a “turning stomach” after sleep disruption compared to US, whereas males showed no detectable increases in negative opioid agonist effects after sleep disruption.

To our knowledge, this is the first study demonstrating that any form of sleep loss attenuates opioid analgesia in humans. This finding extends a prior observational study, which found that sleepy adults exhibited diminished codeine analgesia compared to non-sleepy adults^27^. Our finding that sleep disruption reduced morphine analgesia is also in line with several human quantitative sensory testing experiments indicating that sleep disruption and/or total sleep deprivation impairs pain inhibitory capacity^26,28^, which is mediated, in part, by opioidergic mechanisms^28^. Together, these findings suggest the possibility that sleep disruption may contribute to a vicious dose escalation cycle, whereby poor sleep leads to reduced analgesia, increased pain and subsequent elevation of opioid dose requirement, which may, in turn, further disrupt sleep. At least one clinical study partially supports this hypothesis, demonstrating that one night of poor sleep predicts next day increases in pain and opioid dose among burn injury patients^14^.

The current study successfully translates preclinical investigations which have found that sleep deprivation attenuates morphine’s or MOR agonist’s efficacy to reduce behavioral measures of pain sensitivity in rodents^8–10,29,30^. These and other pre-clinical studies have found that sleep loss alters molecular pathways in cerebral MOR activated anti-nociceptive circuits in the periaqueductal grey, hypothalamus, and mesolimbic structures, providing a neurobiological substrate supporting the current findings. In addition, a prior [^11^C] Carfentanil Positron Emission Tomography (PET) study provides correlational support in humans, demonstrating that short sleep duration was associated with increased MOR availabilty during experimental pain (indicating down regulation of cerebral MOR or diminished release of endogenous opioid peptides) in brain structures associated with pain inhibiton (i.e., anterior cingulate gyrus and dorsolateral preforntal cortex)^31^.

The finding that sleep disruption impairs MOR-based analgesia in humans has major implications for both chronic pain and opioid use disorder (OUD) prevention and management. Sleep complaints impact the vast majority of chronic pain and OUD patients and are increasingly identified as a shared risk factor for both disorders^6,7^. This suggests that highly prevalent sleep disorders, such as insomnia, sleep apnea, and insufficient sleep should be routinely assessed and treated in patients who are candidates for opioid therapy or who struggle with problematic opioid use. The current data also strongly supports the need for clinical research to test whether improved sleep increases opioid analgesic effectiveness, reduces the need for opioids, and facilitates dose reduction among patients on long-term opioid therapy and/or those with OUD undergoing opioid-agonist treatment.

Relatedly, our findings have particular implications for pre-surgical assessment and care. High opioid dosing, perioperatively, is one of the most robust predictors of opioid abuse after surgery. Thus, sleep assessment and interventions prior to surgery may be an important, modifiable approach in preventing not only persistent post-operative pain but also problematic post-operative opioid use^32^. In fact, a recent study has demonstrated that a week of sleep extension, prior to knee or hip replacement surgery, reduced post-operative opioid usage^33^.

In addition to attenuation of opioid analgesia, we also found that sleep disruption diminished the subjective drug effect ratings of “feeling high,” in females, but not males. This notable sex difference complements our additional finding that sleep disruption also increased negative opioid agonist effects (e.g., turning stomach, dry mouth, etc.) in females, but not males. Previous studies have found that females, in general, report greater negative drug effects^25,34^, but we are not aware of any prior studies evaluating whether this effect might be moderated by sleep disturbance. These sex differences in “feeling high” and negative drug effects suggest a possible protective benefit for females relative to males with respect to opioid abuse liability. Sex differences in the rates of substance abuse in the general population typically demonstrate that males have a higher prevalence of substance use disorders, though studies in opioid use disorder are more mixed^35^. Furthermore, the finding that sleep disrupted males demonstrated no significant attenuation in the hedonic, reinforcing properties of morphine (either drug “liking” or “high”) and, no increase in negative opioid effects, but simultaneously experienced diminished analgesic benefit, suggests the intriguing possibility that sleep-disrupted males may be at increased risk for problematic opioid use. Consistent with this possibility, a recent population-based study demonstrated that males with chronic pain were at higher risk than females in progressing to high-dose opioid therapy^36^. It is also important to note that in many circumstances such as cancer-related pain and palliative care, high dose opioids are important necessary options. Our findings suggest that maximizing sleep may help improve analgesia and may help females better tolerate negative drug effects.

Several important caveats should be considered in evaluating these findings. First, the present study was conducted in relatively young, mentally and physically healthy, good sleeper adults, and therefore it remains unclear the extent to which these findings may generalize to older adults or those with chronic sleep disturbance, chronic pain, or OUD. However, given the potential harm associated with administering opioids to at-risk individuals, first establishing these effects in a healthy population is an important and necessary step that will: 1) support carefully conducted studies in at-risk populations and, 2) support prevention studies determining whether treating sleep disturbance improves opioid pain management and reduces problematic outcomes, including abuse and overdose. Second, because we only tested a single morphine dose, it is possible that different patterns of findings could emerge at different doses not tested here. Third, although we were adequately powered to detect two-way interactions (e.g., Sleep Condition × Drug Condition), findings from the three-way interactions examining sex differences should be considered exploratory due to the relatively small sample size for three-way interactions. Lastly, recent studies demonstrate that physical activity levels also serve an important role in pain inhibitory function and mood, especially in women^37,38^. Although the present study did not measure physical activity level, future studies should consider measuring physical activity and evaluate how it may interact with sleep loss on pain and analgesia.

In summary, we found that two nights of experimental sleep disruption attenuates morphine analgesia in healthy adults. Sleep disruption diminished both the subjective drug effects of feeling high and increased negative opioid effects in females, but not males. Given that sleep disturbance is a highly prevalent, modifiable risk factor for chronic pain and OUD, these findings suggest that identifying and targeting sleep disruption may improve outcomes among individuals with chronic pain and those with or at risk for problematic opioid use.

## Methods

### Study design and participants

This study was a mixed methods experiment, including a randomized, double-blind, parallel arm, placebo-controlled component [between subjects factor (morphine versus placebo)], and a cross-over component for sleep condition [within subjects factor (undisturbed sleep versus disturbed sleep)]. The sequence of the cross-over sleep conditions was randomized. After a polysomnographic screening/ adaptation night, eligible participants completed the experimental phase, undergoing two consecutive nights of either US or FA sleep disruption. Participants completed a two-week “washout” interval of habitual sleep in the home environment before completing the opposing sleep condition. The morning after the second night of both US and FA, subjects completed cold pressor pain tolerance testing immediately before and 40 min after a subcutaneous (S.C.) injection of 0.08 mg/kg of morphine or placebo saline solution. We chose the 40-min post-drug injection assessment time point to capture peak pharmacological effects of S.C. injected morphine. Participants remained in the same drug arm under both sleep conditions.

The current study is part of a multi-aimed project. We previously reported results from a smaller cohort focusing on quantitative sensory testing prior to drug injection (baseline), demonstrating that sleep disruption promoted a pain sensitivity profile with central sensitization in a sex-dependent manner^39^. As designed, this prior report did not evaluate morphine analgesia and only using data assessed prior to the morphine/placebo injection. In the present report, we used a larger cohort of participants and assessed cold pressor pain tolerance before and after participants were administered morphine versus placebo.

All participants were recruited from the community using social media platforms, print advertisements, and community fliers. Prior to enrollment, participants were pre-screened to determine eligibility. See Table 3 for inclusion and exclusion criteria. Subjects completed an extensive screening phase to establish eligibility criteria as healthy good sleepers. At Visit 1, participants completed informed consent, structured diagnostic interviews for sleep, psychiatric, and substance use disorders, along with a standard battery of questionnaires. Participants then completed two weeks of electronic sleep and pain diary monitoring to establish normal sleep and pain free status. Screening Visit 2 included a history and physical exam, urinalysis (toxicology and pregnancy screens), and a complete blood count test. Participants who remained eligible were admitted to the clinical research unit (CRU) at Johns Hopkins Bayview Medical Center for Visit 3, which included an additional toxicology screening and a full diagnostic polysomnogram, to rule out occult sleep disorders and provide an adaptation night.

The study was conducted over five days, during two separate inpatient admissions to the Johns Hopkins Bayview Medical Center, Clinical Research Unit (CRU) in Baltimore, MD. The protocol was approved by the Johns Hopkins University School of Medicine Institutional Review Board in compliance with the Declaration of Helsinki and registered with clinicaltrials.gov (NCT01794689; date of registration 20/02/2013). All subjects completed informed consent prior to participation.

### Sample size determination

We could find no prior human studies that investigated the influence of any form of experimental sleep disruption or loss on opioid analgesia. We therefore based our effect size estimate calculations on unpublished data from a randomized double-blind, placebo-controlled study of intramuscularly injected alfentanil [15 mcg/kg], a MOR agonist, in 18 male subjects (nine meeting self-reported criteria for poor sleep). Poor sleeper status diminished alfentanil analgesia (measured similarly to the current study via cold pressor pain testing) with an estimated effect size of d = 1.9. We also considered a prior cross-sectional study which investigated the effects of daytime sleepiness on codeine analgesia (d = 0.9)^27^. Based upon these data, we conducted Monte Carlo simulations, which showed that a sample size N = 80 had 80% power to detect effects of d ≥ 0.7. Therefore, we were sufficiently powered to detect similar effects reported in the literature and in our pilot data with N = 94 (the final sample size of the present study).

### Randomization and masking

Participants were randomized by the study biostatistician to 1 of 4 sequences in collaboration with a licensed, independent investigational pharmacist who was masked to the study aims: (1) Placebo/US first (followed by Placebo/FA two weeks later); (2) Placebo/FA first (followed by Placebo/US two weeks later); (3) Morphine/US first (followed by Morphine/FA two weeks later); or (4) Morphine/FA first (followed by Morphine/US two weeks later. We used a dynamic probabilities algorithm with minimization^40^ to balance groups on sex, body mass index, and age. Neither the pharmacist nor the statistician had any participant contact. The pharmacist prepared the morphine and placebo (saline). Syringe contents were matched on volume and appearance. Injections were administered by nursing staff, who along with the participants were masked to injection contents for the duration of the project.

### Procedures

Eligible participants completed either two nights of US or two nights of FA. A minimum 2-week washout period was maintained between sleep conditions, during which participants continued sleep diary monitoring. At the return visit, after toxicology and pregnancy testing, participants underwent two nights of the opposing sleep condition.

During inpatient CRU visits, subjects were provided a standardized heart healthy diet. Breakfast was served at approximately 7:30, lunch at approximately 12:00, and dinner at about 17:30. Analgesics, caffeine, nicotine, and alcohol were prohibited during the inpatient stay. Subjects remained on the unit at all times, were directly observable by nursing staff, and were not permitted to nap outside of the defined sleep opportunity period between 23:00 and 07:00. Wakefulness during the day was formally documented every 15 min.

Sleep was monitored polysomnographically (Embla N7000, Natus Medical Incorporated) each inpatient night in accordance with standardized acquisition and sleep scoring procedures^41^, using Embla REMLogic Software. The Visit 3 PSG included a full respiratory and electromyography (EMG) procedure to diagnose any sleep disordered breathing and periodic limb movement disorder. The remaining four PSGs conducted during Visits 4 and 5 used an abbreviated procedure without respiratory sensors and tibialis EMGs. Polysomnograms were reviewed and scored by a physician who was board certified in sleep medicine.

FA Condition: The FA sleep disruption procedure involves multiple awakenings and was developed as a model for the pattern of sleep loss most commonly experienced by patients with severe insomnia, including patients with chronic pain and substance use disorders^26,39^. Briefly, an eight-hour sleep opportunity period, starting from lights out, was divided into eight, one-hour intervals. One of the intervals was randomly determined to be a 60-min awakening, during which no sleep was permitted. Each of the remaining seven, 60-min intervals were subdivided into tertiles (three 20-min blocks). A forced 20-min awakening was randomly scheduled to occur in either the 1st, 2nd, or 3rd tertile of each hour. During FA, PSG signals were monitored and nursing staff kept participants awake by having them sit up in bed and turning the lights on. The maximum total sleep time possible was 280 min in this condition.

US Condition: Subjects slept undisturbed during an 8-h (480 min) sleep opportunity.

The morning after the second night of both sleep conditions, participants had a light breakfast, and completed the Psychomotor Vigilance Task (PVT) at approximately 10:00 and then completed quantitative sensory testing (QST) battery, including mechanical temporal summation, heat and pressure pain thresholds and tests of heat-capsaicin pain (data reported previously)^39^. At approximately 11:25, subjects completed a cold pressor tolerance test immediately prior to, a S.C. injection of 0.08 mg/kg of morphine or placebo saline solution. Then, 40 min after the drug injection, post-drug administration QST was conducted approximately at 12:10. Note that the pre-registered primary outcome of the present study was the analgesia index based upon changes in the pre- and post-drug cold pressor tolerance score. The Online Supplement Fig. 1 describes the QST protocol in greater detail.

The PVT is a validated behavioral performance measure of sustained attention shown to be highly be sensitive to sleep deprivation^42^. We used the 10-min version as a manipulation check and objective index of sleepiness^43^. The PVT was conducted on a laptop computer with a 15-inch screen that had been calibrated using the Performance Assessment Calibration EngineTM (Pulsar Informatics, Inc.) to ensure high precision and accuracy. Participants were instructed to focus on a rectangular box in the center of the computer screen and press the space bar as soon as they observed a yellow counter appear inside the box. Pressing the space bar stopped the counter, and the corresponding reaction time (RT) appeared on the screen for one second. Inter-stimulus interval varied between 2 and 10 s. Participants were instructed to avoid pressing the space bar prior to the start of the counter; any such instances were coded as lapses. We used the reciprocal response time (mean 1/RT in ms) and lapses as the primary indices of PVT performance.

### Measures

#### Primary outcome: cold pressor pain tolerance testing (cpt)/analgesia index

The cold pressor test (CPT) is specifically validated to evaluate the analgesic effects of opioids^44^ and therefore was the primary, a priori analgesia outcome. Participants immersed their hand up to their wrist in a circulating, cold water bath (4 °C) [Thermo Electron Corporation, Neslab RTE 17], up to an un-informed 5-min maximum time limit. Participants were instructed to keep their hand in the water for as long as possible, until the sensations become intolerable. To create an analgesia index as the primary outcome, based upon a previous study^9^, a change score of hand withdrawal latency (HWL) was computed by subtracting CPT1 (time point 1—prior to drug administration) from CPT2 (time point 2–40-min post drug administration).

#### Secondary outcomes: VAS measurement of drug “liking,” feeling “high,” and drug adjective checklist of negative opioid effects

To assess hedonic response to morphine, participants completed standard visual analog scales (VAS) for the subjective drug effects, “How high are you?” and “Do you like the drug?”, 30-min following morphine or placebo administration. Participants drew a mark on a printed 100-mm line corresponding to a rated scale of 0 (“None”) to 100 (“Extremely”)^45^. The FDA recognizes peak effect scores for “liking” and “high” as principal outcomes in abuse liability research^23,24^. We therefore included these as secondary outcomes for this investigation. Additionally, participants completed the opioid agonist and withdrawal checklist scale to evaluate the severity of negative drug effects associated with opioid administration^46^. Ratings were made on a Likert scale of 1 (not at all) to 5 (extremely). The five negatively valenced opioid agonist adjectives in the checklist were summed to comprise the negative opioid agonist effect subscale. These items were: “heavy or sluggish feeling,” “dry mouth,” “skin itchy,” “turning stomach,” and “nervous.” Opioid agonist withdrawal checklist items were not used because subjects did not have physical dependence on opioids and they were assessed during peak morphine effects.

### Covariates

Model covariates were selected based on evidence consistently linking these factors with pain sensitivity and/or analgesia^47–50^; these covariates were age, race (trichotomized to White vs. Black vs. Other Race), body mass index (BMI), systolic and diastolic blood pressure at baseline, and menstrual phase (either menstruating or pre-menstrual was categorized as 1, while any other phase was categorized as 0). Of note, to estimate menstrual phase, participants kept standard daily sleep diaries for two weeks before and during each inpatient stay. For each diary entry, female participants were asked the typical length of their menstrual cycle, if they were currently menstruating, and if their period started or ended that day. Based upon these data, we estimated each female subjects’ menstrual cycle. For each inpatient day if a female participant was either menstruating or in the pre-menstrual phase, the associated menstrual variable was labeled as a 1. If a female subject was not in these two phases, then they were categorized as 0.

### Statistical analyses

The distribution of each outcome variable was inspected for normality. As all the outcomes demonstrated skewness > 1, they were log-transformed using natural log. Given the nested data structure (i.e., repeated outcome assessments at each sleep condition) along with our aim to examine both between- and within-subject sources of variance, we conducted linear mixed-effects modeling using SPSS Version 24. We chose linear mixed-effects modeling rather than the repeated-measures ANOVA because it is robust to homoscedasticity, sphericity violations, and missing data.

Analyses of the primary and secondary outcomes were based upon the intention-to-treat (ITT) principle. All models included the 2 × 2 (two-way) interaction between sleep conditions (US vs. FA) and drug conditions (morphine vs. placebo) in evaluation of primary and secondary outcomes. Age, race, menstrual phase, BMI, and baseline blood pressure measures were included as covariates in all models. In addition, for the primary outcome (analgesia index) model, we controlled the baseline HWL score because results of change scores (i.e., analgesia index) can be influenced by the baseline levels of the outcome. We conducted planned pairwise comparisons to evaluate whether the effect of morphine differed from placebo under each sleep condition. Sidak-correction was used to balance Type I and Type II errors. A two-sided *p* < 0.05 was considered statistically significant.

In addition to analyses of primary and secondary outcomes, we explored the possible moderating role of sex because: (1) we found in our previous study that sex moderated the effect of sleep disruption on central sensitization^39^; (2) males have higher rates of substance abuse^35^, and (3) some data suggest that females may be more responsive to opioid analgesia, but have more negative side effects^25,34^. To explore possible sex effects on the outcomes, we tested the three-way interaction effect (Sleep Condition × Drug Condition × Sex), as well as the Sleep Condition × Sex interaction if the three-way interaction was not statistically significant.

## Supplementary information

Supplementary Information.

Supplementary Information.

Supplementary Information.

## Data Availability

The trial dataset and full trial protocol will be available to appropriate academic parties upon request to the corresponding author, in accordance with the data sharing policies of Johns Hopkins School of Medicine, with input from the investigator group where applicable, subject to submission of a suitable study protocol and analysis plan, on publication of all initial trial results.
