# Supplementary material for: Experimental sleep disruption attenuates morphine analgesia: findings from a randomized trial and implications for the opioid abuse epidemic
Source: Sci Rep. 2020 Nov 18;10:20121. doi: 10.1038/s41598-020-76934-1 (PMC7674501; doi:10.1038/s41598-020-76934-1)
Supplement: Supplementary file 1 — Supplementary Information. [file 41598_2020_76934_MOESM1_ESM.docx]

**Experimental sleep disruption attenuates morphine analgesia:**

**Findings from a randomized trial and implications for the opioid abuse epidemic**

Michael T. Smith, PhD^1^, Chung Jung Mun, PhD^1^, Bethany Remeniuk, PhD^1^, Patrick H. Finan, PhD^1^,

Claudia M. Campbell, PhD^1^, Luis F. Buenaver, PhD^1^, Mercedes Robinson, BS^2^, Brook Fulton, BA^1^,

David Andrew Tompkins, MD^3^, Jean-Michel Tremblay, PhD^4^, Eric C. Strain, MD^1^, and

Michael R. Irwin, MD^5,6^

^1^Department of Psychiatry and Behavioral Sciences, Johns Hopkins University, School of Medicine, Baltimore, MD, USA 21224

^2^Virginia Tech Carilion School of Medicine, Roanoke, VA, USA 24016

^3^Department of Psychiatry, UCSF School of Medicine, San Francisco, CA, USA 94110

^4^Edgybees Inc., Gaithersburg, MD, USA 20878

^5^Cousins Center for Psychoneuroimmunology, UCLA Semel Institute for Neuroscience and Human Behavior, Los Angeles, CA, USA 90024

^6^Department of Psychiatry and Biobehavioral Sciences, David Geffen School of Medicine at UCLA, Los Angeles, CA, USA 90095

**Corresponding Author:**

Michael T. Smith, Ph.D.

Professor of Psychiatry and Behavioral Sciences and Neurology

Johns Hopkins School of Medicine,

Division of Behavioral Medicine

5510 Nathan Shock Drive, Suite 100, Baltimore, MD 21225

P: 410-550-7000

E: [msmith62@jhmi.edu](mailto:msmith62@jhmi.edu)

**Online Supplementary Materials**

**Online Supplement Table 1.** Sleep condition manipulation check on sleep continuity and sleep architecture.

| **Sleep Continuity, M(SD)** | **US_1_** | **US_2_** | **FA_1_** | **FA_2_** | **Mean Difference**  **(FA_1,2_) – (US_1,2_)** |
| --- | --- | --- | --- | --- | --- |
| SL | 18.58 (30.15) | 18.43 (20.97) | 31.06 (31.23) | 19.21 (21. 62) | 6.81 (2.92)* |
| WASO | 24.20 (25.47) | 23.20 (29.86) | 215.71 (48.50) | 195.77 (33.37) | 182.33 (3.94)*** |
| TST | 436.44 (38.99) | 434.83 (49.81) | 232.45 (43.52) | 264.49 (28.63) | -187.66 (4.62)*** |
| SE% | 91.7 (8.00) | 91.10 (7.92) | 48.50 (9.01) | 55.16 (5.91) | -39.35 (.89)*** |
|  |  |  |  |  |  |
| **Sleep Architecture, M(SD)** | **US_1_** | **US_2_** | **FA_1_** | **FA_2_** | **Mean Difference**  **(FA_1,2_) – (US_1,2_)** |
| Stage 1 | 18.22 (18.52) | 15.98 (9.95) | 22.96 (12.44) | 19.15 (10.63) | 4.00 (1.46)** |
| Stage 2 | 215.81 (49.84) | 207.12 (49.66) | 113.25 (27.08) | 118.59 (23.80) | -95.68 (4.30)*** |
| Stage 3 (SWS) | 109.21 (47.44) | 112.02 (43.06) | 62.39 (26.02) | 74.22 (25.10) | -42.47 (4.02)*** |
| REM | 93.20 (28.30) | 99.71 (27.24) | 33.86 (20.14) | 52.52 (21.24) | -53.51 (2.77)*** |
| REM Latency | 94.47 (43.49) | 78.39 (36.79) | 180.40 (102.85) | 134.29 (82.47) | 71.10 (8.12)*** |

**Note.** Values shown represent mean (SD) in minutes.

**Key**: US_1,2_=the average of Undisturbed Sleep Nights 1 and 2; FA_1,2_ = the average of Forced Awakenings Nights 1 and 2; SL = sleep onset latency; WASO = wake after sleep onset time; TST= total sleep time; SE% = sleep efficiency percentage (TST/time in bed); Stages 1-3 = non-rapid eye movement stages 1-3; SWS = slow wave sleep; REM = Stage rapid eye movement sleep. *p<.05; **p<.01; ***p<.001.

**Online Supplement Table 2.** Means and standard deviations of pre- and post-injection hand withdrawal latency by conditions.

| **Drug Condition** | **Sleep Condition** | **Pre-injection**  **Mean Raw HWL (SD)** | **Post-injection**  **Mean Raw HWL (SD)** | **Pre-injection**  **Mean Log HWL (SD)** | **Post-injection**  **Mean Log HWL (SD)** |
| --- | --- | --- | --- | --- | --- |
| Placebo | US | 96.1 (113) | 91.1 (114) | 3.90 (1.15) | 3.77 (1.19) |
| Placebo | FA | 92.8 (119) | 95.2 (122) | 3.75 (1.22) | 3.77 (1.21) |
| Morphine | US | 110 (125) | 132 (127) | 3.94 (1.29) | 4.22 (1.18) |
| Morphine | FA | 110 (123) | 123 (123) | 3.96 (1.28) | 4.29 (1.15) |

**Note.** Values shown represent mean (SD) in seconds

**Key**: US = Undisturbed Sleep Condition, FA = Forced Awakenings Sleep Disruption Condition, HWL = hand withdrawal latency from cold pressor tolerance test.

**Online Supplement Table 3.** Reported adverse events

| Total Adverse Events | 24 |
| --- | --- |
| Sex |  |
| Female | 18 (75%) |
| Male | 6 (25%) |
| Sleep Condition |  |
| US | 10 (41.7%) |
| FA | 14 (58.3%) |
| Participants with AE at Both Sleep Conditions | 7 out of 17 |
| Drug |  |
| Morphine | 22 (91.7%) |
| Placebo | 2 (8.3%) |
| Characteristics of AE |  |
| Nausea | 20 |
| Vomiting | 15 |
| Low Blood Pressure | 4 |
| Fainting | 1 |
| Headache | 1 |
| Pain | 1 |
| Medical Issue Prior to Inpatient Stay | 1 |

**Note**. Values represent mean (percent of total) for participants who reported experiencing an adverse event. This excludes individuals who were removed from the study.

**Online Supplement Figure 1.** Quantitative sensory testing protocol


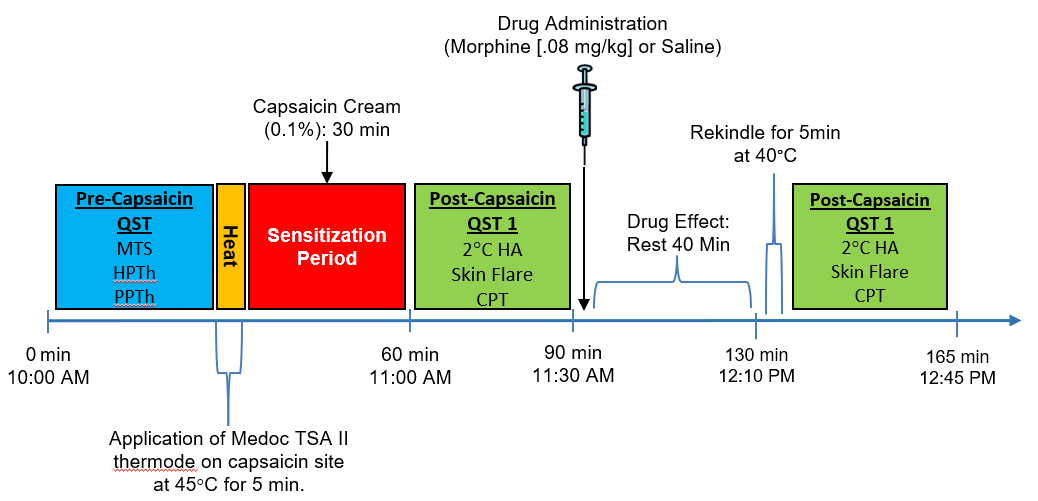


*Note*. Following two nights of FA or US, participants underwent daytime quantitative sensory testing. Pre-capsaicin quantitative sensory testing measures included mechanical temporal summation (MTS), heat pain threshold (HPTh), and pressure pain threshold (PPTh). After 5 min of 45°C heat application via Medoc ATS II thermode to a predetermined location on the medial forearm, 0.1% capsaicin cream was applied and allowed to rest 30 min. Following removal of the capsaicin cream, post-capsaicin assessments included secondary hyperalgesia (2° HA), skin flare, and cold pressor pain tolerance testing (CPT). Note that the pre-registered primary outcome of the present study was the analgesia index based upon changes in pre- and post-drug CPT.
