# Supplementary material for: Experimental sleep disruption attenuates morphine analgesia: findings from a randomized trial and implications for the opioid abuse epidemic
Source: Sci Rep. 2020 Nov 18;10:20121. doi: 10.1038/s41598-020-76934-1 (PMC7674501; doi:10.1038/s41598-020-76934-1)
Supplement: Supplementary file 3 — Supplementary Information. [file 41598_2020_76934_MOESM3_ESM.pdf]

COVID-19 is an emerging, rapidly evolving situation.
Get the latest public health information from CDC: https://www.coronavirus.gov.
Get the latest research information from NIH: https://www.nih.gov/coronavirus.

Mechanisms of Sleep Disruption Hyperalgesia (ESP2)

The safety and scientific validity of this study is the responsibility of the study sponsor and investigators. Listing a study does not mean it has been evaluated by the U.S. Federal Government. Read our disclaimer for details.

ClinicalTrials.gov Identifier: NCT01794689

Recruitment Status : Completed
First Posted : February 20, 2013
Results First Posted : February 22, 2019
Last Update Posted : August 2, 2019

Sponsor:
Johns Hopkins University

Collaborator:
National Institute on Drug Abuse (NIDA)

Information provided by (Responsible Party):
Johns Hopkins University

Study Description

Go to

Brief Summary:

Twenty percent of Americans suffer from chronic pain. Sleep disturbance is similarly prevalent and among the most common and disabling neurobehavioral problems associated with chronic pain. This research is designed to evaluate the effects of disrupted sleep patterns on mood, inflammation, the perception of pain, and pain relief. This study will help researchers understand the relationship between sleep and pain, and how sleep disturbance might influence chronic pain conditions.

Condition or disease	Intervention/treatment	Phase
Sleep Deprivation<br>Pain	Drug: Morphine<br>Drug: Saline Placebo<br>Behavioral: Forced Awakenings<br>Behavioral: Uninterrupted Sleep	Not Applicable

Detailed Description:

This research is being conducted in order to evaluate the effects of disrupted sleep patterns on mood, inflammation, the perception of pain, and pain relief. This study will help researchers understand the relationship between sleep and pain, and how sleep disturbance might influence chronic pain conditions. Healthy participants will undergo baseline sleep and sleep disruption conditions. Following undisturbed sleep and sleep disruption conditions, sensitivity to pain and analgesic response (via morphine or placebo administration) will be assessed using a heat-capsaicin pain model.

This study will be conducted in 2 major parts-3 screening visits (2 outpatient and 1 inpatient) and 2 experimental inpatient visits. Part 1 of the study will involve a 1-week screening period. This will involve two separate screening visits lasting about 2 hours each. At Screening Visit 1, participants will complete questionnaires, an interview, and undergo toxicology screening. At Screening Visit 2, participants will complete questionnaires, undergo a physical exam, and be familiarized with pain testing procedures. At Screening Visit 3, participants will undergo an inpatient sleep study.

Part 2 will involve two different inpatient admissions. The two admissions will be separated by at least two weeks. During each of the admissions, participants' sleep will be studied at night. The first admission will begin immediately following the overnight sleep study in Screening Visit 3. One of the admissions will be for one night and the other admission will be for three nights. For the one night admission, participants will sleep undisturbed for an 8-hour period. For the three night admission, participants will undergo sleep disruption for two nights in a row. On the third night, participants will be allowed to sleep undisturbed for 8 hours for recovery.

During both inpatient admissions, pain testing procedures will be completed that will last approximately 5 hours during the day. During testing, small amounts of blood will be drawn for analysis. Participants will be randomly assigned to two groups. Group A will be given a standard dose of morphine during pain testing. Group B will be given a placebo during pain testing.

Study Design

Go to

Study Type :	Interventional (Clinical Trial)
Actual Enrollment :	100 participants
Allocation:	Randomized
Intervention Model:	Crossover Assignment
Masking:	Double (Participant, Care Provider)
Primary Purpose:	Basic Science
Official Title:	Mechanisms of Sleep Disruption Hyperalgesia
Actual Study Start Date :	May 2013
Actual Primary Completion Date :	January 2018
Actual Study Completion Date :	March 12, 2019

Arms and Interventions

Go to

Arm	Intervention/treatment
Experimental: Morphine US then Morphine FA<br>Participants randomized to receive Morphine and the Uninterrupted Sleep (US) condition first. After a polysomnography (PSG) screening night, participants were randomized to receive two consecutive nights of uninterrupted sleep (US). With a minimum of a two	Drug: Morphine<br>0.08mg/kg will be administered to participants randomly assigned to receive the drug via IV bolus during each quantitative sensory testing session (after one night of uninterrupted sleep and after 2 nights of forced awakenings).

Arm	Intervention/treatment
week washout period, participants then completed the opposing sleep condition of two nights of forced awakenings (FA). They will receive the Morphine injection (0.08mg/kg) via IV bolus over 30 seconds during each experimental quantitative sensory testing session that occurs after the US and FA sleep conditions are completed.	Behavioral: Forced Awakenings<br>Participants will be awakened each hour during an 8 hour sleep opportunity period. One of the awakenings is for 60 minutes and randomly determined. The other 7 awakenings are for 20 minutes each, and are randomly scheduled to occur in either the first second or third tertile of each hour. The maximum total sleep time a participant will receive is 280 minutes.<br><br>Behavioral: Uninterrupted Sleep<br>Participants will receive an 8 hour period of undisturbed sleep
Placebo Comparator: Placebo US then Placebo FA<br>Participants randomized to receive the saline placebo and the Uninterrupted Sleep (US) condition first. After a polysomnography (PSG) screening night, participants were randomized to receive two consecutive nights of uninterrupted sleep (US). With a minimum of a two week washout period, participants then completed the opposing sleep condition of two nights of forced awakenings (FA). They will receive the injection via IV bolus over 30 seconds during each experimental quantitative sensory testing session that occurs after the US and FA sleep conditions are completed.	Drug: Saline Placebo<br>Saline Placebo will administered to participants randomly assigned to receive the placebo via IV bolus during each quantitative sensory testing session (after one night of uninterrupted sleep and after 2 nights of forced awakenings).<br><br>Behavioral: Forced Awakenings<br>Participants will be awakened each hour during an 8 hour sleep opportunity period. One of the awakenings is for 60 minutes and randomly determined. The other 7 awakenings are for 20 minutes each, and are randomly scheduled to occur in either the first second or third tertile of each hour. The maximum total

Arm	Intervention/treatment
	sleep time a participant will receive is 280 minutes.<br><br>Behavioral: Uninterrupted Sleep<br>Participants will receive an 8 hour period of undisturbed sleep
Experimental: Morphine FA then Morphine US<br>Participants randomized to receive Morphine and the Forced Awakenings (FA) condition first. After a polysomnography (PSG) screening night, participants were randomized to receive two consecutive nights of forced awakenings (FA). With a minimum of a two week washout period, participants then completed the opposing sleep condition of two nights of uninterrupted sleep (US). They will receive the Morphine injection (0.08mg/kg) via IV bolus over 30 seconds during each experimental quantitative sensory testing session that occurs after the FA and US sleep conditions are completed.	Drug: Morphine<br>0.08mg/kg will be administered to participants randomly assigned to receive the drug via IV bolus during each quantitative sensory testing session (after one night of uninterrupted sleep and after 2 nights of forced awakenings).<br><br>Behavioral: Forced Awakenings<br>Participants will be awakened each hour during an 8 hour sleep opportunity period. One of the awakenings is for 60 minutes and randomly determined. The other 7 awakenings are for 20 minutes each, and are randomly scheduled to occur in either the first second or third tertile of each hour. The maximum total sleep time a participant will receive is 280 minutes.<br><br>Behavioral: Uninterrupted Sleep<br>Participants will receive an 8 hour period of undisturbed sleep
Placebo Comparator: Placebo FA then Placebo US<br>Participants randomized to receive the saline placebo and the Forced	Drug: Saline Placebo<br>Saline Placebo will administered to participants randomly assigned to receive the placebo via IV bolus during each

Arm	Intervention/treatment
Awakenings (FA) condition first. After a polysomnography (PSG) screening night, participants were randomized to receive two consecutive nights of forced awakenings (FA). With a minimum of a two week washout period, participants then completed the opposing sleep condition of two nights of uninterrupted sleep (US). They will receive the injection via IV bolus over 30 seconds during each experimental quantitative sensory testing session that occurs after the FA and US sleep conditions are completed.	quantitative sensory testing session (after one night of uninterrupted sleep and after 2 nights of forced awakenings).<br><br>Behavioral: Forced Awakenings<br>Participants will be awakened each hour during an 8 hour sleep opportunity period. One of the awakenings is for 60 minutes and randomly determined. The other 7 awakenings are for 20 minutes each, and are randomly scheduled to occur in either the first second or third tertile of each hour. The maximum total sleep time a participant will receive is 280 minutes.<br><br>Behavioral: Uninterrupted Sleep<br>Participants will receive an 8 hour period of undisturbed sleep

Outcome Measures

Go to

Primary Outcome Measures :

1. Spinal Sensitization as Assessed by Area of Secondary Hyperalgesia (2HA) After Two Nights of Uninterrupted Sleep and Two Nights of 8 Forced Awakenings
   [Time Frame: Next day during quantitative sensory testing after 2 nights of forced awakenings or uninterrupted sleep]

   The area of secondary hyperalgesia (2HA) to mechanical stimulation was quantified by stimulating along eight linear paths near the capsaicin treated site using a 15 gram nonpainful von Frey filament. Stimulation occurred until the participant reported a change in sensation from which a border was marked on the skin. The degree of 2HA was assessed by measuring the total surface area (mm^2) of the marked borders. Data were collapsed by group to analyze the effects of FA vs US, irrespective of

randomization group. Our Primary Secondary Hyperalgesia outcome was measured prior to injection of either morphine or placebo.

Secondary Outcome Measures :

1. Opioid Analgesia as Assessed by Analgesia Index (Seconds) [Time Frame: Next day after 2 nights of forced awakenings or uninterrupted sleep]

   After 2 nights of forced awakenings and after two nights of uninterrupted sleep, opioid analgesia will be assessed by an analgesia index. The analgesia index is calculated using withdrawal latency during cold pressor testing (lasting maximum of 300 seconds). Cold Pressor Testing is done before and after morphine or placebo injection. The difference in withdrawal time before and after morphine or placebo injection is the analgesia index with a minimum score of -300 seconds and maximum score of 300 seconds. These data were log transformed. Mean analgesia index was then calculated for each group. Higher means represent greater analgesia.

2. Mean Change in Percentage of Peripheral Blood Mononuclear Cells Expressing Interleukin-6 After LPS Stimulation [Time Frame: Next day after 2 nights of forced awakenings or uninterrupted sleep, every 60 minutes up to 7 hours]

   After 2 nights of forced awakenings, and two nights of uninterrupted sleep, blood is drawn (approximately every 60 minutes) during quantitative sensory testing; 4 hours pre-morphine/placebo administration and 2 hours post-morphine/placebo administration to examine markers of inflammation. Two blood samples for each participant are analyzed at 7 separate time points. The marker of inflammation assessed is the number of peripheral blood mononuclear cells expressing Interleukin-6 (IL-6), and the outcome measure represents the mean change in IL-6 levels pre and post stimulation with lipopolysaccharide (LPS). Cellular IL-6 expression was was quantified via flow cytometry.

Other Outcome Measures:

1. Total Sleep Time [Time Frame: Next day during quantitative sensory testing after 2 nights of forced awakenings or uninterrupted sleep.]

The degree of sleep deprivation will be assessed by total sleep time (TST) in minutes during the uninterrupted sleep condition compared to the forced awakenings conditions.

Eligibility Criteria

Go to

Information from the National Library of Medicine

Choosing to participate in a study is an important personal decision. Talk with your doctor and family members or friends about deciding to join a study. To learn more about this study, you or your doctor may contact the study research staff using the contacts provided below. For general information, Learn About Clinical Studies.

Ages Eligible for Study:	18 Years to 48 Years (Adult)
Sexes Eligible for Study:	All
Accepts Healthy Volunteers:	Yes

Criteria

Inclusion Criteria:

- Healthy
- Age 18-48
- Meets Research Diagnostic Criteria for Normal Sleepers
- Stable sleep phase within 21:00 and 08:00
- Total sleep time between 6.5 and 8.5 hours per night
- Sleep efficiency ≥85%
- Epworth Sleepiness Scale Score <10
- Non-smoker/non-nicotine users
- Low Caffeine Users (≤2 cups per day)

Exclusion Criteria:

- Body Mass Index ≥35
- Lifetime history of chronic pain (>6 months)
- Acute pain
- Significant medical or psychiatric morbidity within 6 months
- Lifetime history of bipolar disorder, psychotic disorder, serious recurrent major depression, serious post-traumatic stress disorder, or seizure disorder
- Respiratory, hepatic, renal, or cardiac conditions that would contraindicate opioid use
- Lifetime history of alcohol or substance abuse or dependence
- Lifetime history of opioid use >36 doses or >7 days of consecutive use
- Prior adverse reaction to general anesthetics, opioids, or capsaicin
- Clinically significant abnormal complete blood count or comprehensive metabolic profile
- Positive toxicology screen for opioids or recreational drugs
- Pregnant or lactating women
- Significant pre-admission psychological distress (T-scores >64 on the Brief Symptom Inventory Global Scales)
- Significant lifetime history of serious head injury that is determined to influence pain processing or sleep systems

Contacts and Locations

Go to

Information from the National Library of Medicine NIH NLM

To learn more about this study, you or your doctor may contact the study research staff using the contact information provided by the sponsor.

Please refer to this study by its ClinicalTrials.gov identifier (NCT number): ***NCT01794689***

Locations

United States, Maryland

Johns Hopkins University Bayview Medical Center
Baltimore, Maryland, United States, 21224

Sponsors and Collaborators

Johns Hopkins University

National Institute on Drug Abuse (NIDA)

Investigators

Principal Investigator: Michael Smith, Ph.D Johns Hopkins University

Study Documents (Full-Text)

Documents provided by Johns Hopkins University:

Study Protocol and Statistical Analysis Plan [PDF] August 30, 2016

More Information

Go to

Responsible Party:	Johns Hopkins University
ClinicalTrials.gov Identifier:	NCT01794689 History of Changes
Other Study ID Numbers:	NA_00071465
	1R01DA032922-01 (U.S. NIH Grant/Contract)
First Posted:	February 20, 2013 Key Record Dates
Results First Posted:	February 22, 2019
Last Update Posted:	August 2, 2019
Last Verified:	August 2019

Additional relevant MeSH terms:

Hyperalgesia	Mental Disorders
Sleep Deprivation	Morphine
Somatosensory Disorders	Analgesics, Opioid
Sensation Disorders	Narcotics
Neurologic Manifestations	Central Nervous System Depressants
Nervous System Diseases	Physiological Effects of Drugs
Signs and Symptoms	Analgesics

Dyssomnias	Sensory System Agents
Sleep Wake Disorders	Peripheral Nervous System Agents
